# Knowledge, Attitudes, and Self-Reported Practices Regarding Modern Adhesive Systems Among Romanian Dentists: A Cross-Sectional Survey

**DOI:** 10.3390/jfb17050243

**Published:** 2026-05-12

**Authors:** Razvan Flueras, Ramona Amina Popovici, Aniela-Roxana Nodiți-Cuc, Andreea-Mihaela Kiș, Diana Marian, Dana Emanuela Pitic (Cot), Laria-Maria Trusculescu, Adina Feher, Andreea Salcudean, Aura Mara Bodnar, Ana Gabriela Seni, Norina Consuela Forna, Iustin Olariu

**Affiliations:** 1 Doctoral School, “Victor Babeș” University of Medicine and Pharmacy Timișoara, 300041 Timișoara, Romania; razvan.flueras@umft.ro (R.F.); dana.pitic@umft.ro (D.E.P.); adina.feher@umft.ro (A.F.); aura-mara.ardelean@umft.ro (A.M.B.); 2 Department of Dentistry, Faculty of Dentistry, “Vasile Goldiş” Western University of Arad, 94-96 Revoluţiei Blvd., 310025 Arad, Romania; olariu.iustin@uvvg.ro; 3 Department of Management and Communication in Dental Medicine, Department I, Faculty of Dental Medicine, “Victor Babeș” University of Medicine and Pharmacy of Timișoara, 300041 Timișoara, Romania; ramona.popovici@umft.ro (R.A.P.); laria.trusculescu@umft.ro (L.-M.T.); 4 Department of Surgical Oncology, ‘Carol Davila’ University of Medicine and Pharmacy, 020021 Bucharest, Romania; aniela.noditi@umfcd.ro; 5 Department of Ethics and Social Sciences, “George Emil Palade” University of Medicine, Pharmacy, Science and Technology of Târgu Mureș, 540139 Târgu Mureș, Romania; andreea.salcudean@umfst.ro; 6 Doctoral School, “George Emil Palade” University of Medicine, Pharmacy, Science and Technology of Târgu Mureș, 540139 Târgu Mureș, Romania; gabriela.seni@umfst.ro; 7 Department of Implantology, Removable Prostheses and Dental Prostheses Technology, Faculty of Dental Medicine, “Grigore T. Popa” University of Medicine and Pharmacy, 700115 Iasi, Romania; norina.forna@umfiasi.ro

**Keywords:** adhesive dentistry, universal adhesives, cross-sectional survey, knowledge-practice gap, continuing dental education, 10-MDP, Romanian dentists

## Abstract

Background: The rapid evolution of dental adhesive systems presents both opportunities and challenges for clinical practice, particularly regarding the translation of emerging evidence into routine use. Aim: This study aimed to assess the knowledge, attitudes, and self-reported practices related to modern adhesive systems among Romanian dentists and to explore factors associated with their clinical decision-making. Materials and Methods: An observational cross-sectional study was conducted between November 2025 and February 2026 using a 115-item online questionnaire. A convenience sample of 372 Romanian dentists participated. Statistical analysis included descriptive statistics, chi-square and Kruskal–Wallis tests, and multivariate models (multiple linear regression, binary logistic regression, and multinomial logistic regression). Internal consistency of the knowledge scale was assessed using Cronbach’s alpha. Results: The knowledge scale demonstrated good reliability (Cronbach’s α = 0.873). Although 68.0% of respondents reported familiarity with universal adhesives, a discrepancy between awareness and reported clinical application was observed for several key concepts, including MMP inhibitors and hydrolytic stability. Notably, 14.8% of participants were unaware whether their preferred adhesive system contained 10-MDP. Continuing education frequency was the only independent predictor of higher knowledge scores (β = 1.63, *p* = 0.024), while greater clinical experience was inversely associated with rubber dam use (OR = 0.550, *p* = 0.024). Conclusions: The findings suggest a discrepancy between theoretical knowledge and the clinical implementation of modern adhesive concepts. Structured continuing education plays a critical role in improving knowledge and may help bridge this gap in clinical practice.

## 1. Introduction

Adhesive technologies have fundamentally transformed restorative dentistry. Since the introduction of Buonocore’s pioneering acid-etch technique in 1955, adhesive systems have progressively improved bond strength, simplified clinical workflows, and significantly enhanced the performance and longevity of both direct and indirect restorations [1]. The shift from etch-and-rinse (ER) and self-etch (SE) systems to universal, or multi-mode, adhesives allows clinicians to choose the best etching approach for a specific clinical circumstance [2,3]. Universal adhesives, introduced around 2011, are characterised by their versatility and simplified application. Their formulation, often containing the functional monomer 10-methacryloyloxydecyl dihydrogen phosphate (10-MDP), allows for reliable chemical bonding to hydroxyapatite in enamel and dentin, as well as to various restorative substrates, such as zirconia and metal alloys [4,5,6].

One of the most important properties of universal adhesives is their true multi-mode applicability, as they can be used effectively in ER, selective-etch, and SE protocols, offering substantial clinical flexibility and supporting individualised decision-making according to substrate characteristics and lesion type [7,8]. This has streamlined procedures and expanded the indications for adhesive techniques. Moreover, recent evidence has further highlighted the critical importance of chemical interactions at the dentin–material interface, demonstrating that modern bioactive endodontic sealers may promote elemental deposition and enhance dentin bioactivity, thereby supporting the long-term stability and biological performance of adhesive and restorative treatments [9]. In addition, recent advances in antibacterial and bioregenerative nanomaterials have further expanded the translational potential of restorative and adhesive dentistry, emphasising the role of bioactive material design in promoting tissue integration, microbial control, and long-term clinical success [10].

From a biomaterials perspective, contemporary adhesive systems should no longer be regarded solely as luting or bonding agents, but as functional biointerfaces that can modulate the interactions between restorative materials and dental tissues. The incorporation of functional monomers such as 10-MDP, bioactive glass particles, antibacterial nanofillers, and ion-releasing components has transformed adhesive materials into active therapeutic platforms with potential implications for tissue preservation, remineralisation, and long-term interface stability. In the broader context of oral and maxillofacial reconstruction, these biomaterial-driven approaches contribute not only to the longevity of restorative treatments but also to the biological integration of prosthetic, endodontic, and regenerative treatments. Therefore, clinicians’ understanding of the composition, biological behaviour, and evidence-based clinical indications of these materials is essential for successful translational application in modern oral rehabilitation.

Earlier in vitro studies have demonstrated that chemical treatments applied to the enamel surface can influence its morphological integrity and, consequently, affect adhesive performance [11]. However, achieving optimal clinical outcomes with modern adhesive systems requires not only an understanding of surface interactions but also a comprehensive knowledge of material composition, mechanisms of action, and protocol-specific application strategies [12].

Concepts such as the role of matrix metalloproteinase (MMP) inhibitors in preserving the hybrid layer, the importance of hydrolytic stability for bond durability, and the principles of immediate dentin sealing (IDS) are critical for clinical success [13,14,15]. Despite the growing body of scientific evidence supporting these approaches, a gap often exists between evidence-based recommendations and their consistent implementation in daily clinical practice. This gap between awareness and clinical application may be influenced by multiple factors, including the quality of undergraduate education, access to reliable continuing professional development, the impact of dental manufacturers’ marketing, and individual clinical experience [16,17].

Although valuable, this study was limited in scope and did not examine the underlying knowledge or the variables driving clinicians’ decisions. The mechanical performance of restorative materials, including their ability to dissipate energy and structurally adapt under functional loading, plays an important role in predicting long-term clinical success [18]. In addition to mechanical behaviour, the biological compatibility of restorative materials remains a fundamental determinant of clinical success, as previous studies have demonstrated the importance of biocompatibility in maintaining tissue integration and minimising adverse pulpal or periapical responses [19].

A recent study by Olariu et al. (2024) in Romania provided preliminary insights into dentists’ preferences for adhesive systems, indicating a high prevalence of ER procedures and a high frequency of universal adhesive use [20].

The present study aims to address the knowledge-practice gap identified in previous research by conducting a more comprehensive, granular analysis using a larger, more detailed dataset. Specifically, this study seeks to explore factors influencing Romanian dentists’ use of modern adhesive technologies, thereby providing actionable insights to improve alignment between evidence-based recommendations and clinical practice.

The primary objectives of this cross-sectional study were threefold: (1) to assess the current level of knowledge among Romanian dentists regarding modern concepts in adhesive dentistry; (2) to identify their self-reported clinical practices and preferred adhesive strategies; and (3) to analyze the potential associations between dentists’ demographic and professional characteristics (such as clinical experience and primary sources of information) and their knowledge and clinical choices. We hypothesised that a significant gap exists between the awareness of modern adhesive principles and their consistent application in clinical practice.

## 2. Materials and Methods

### 2.1. Study Design and Participants

An observational, cross-sectional study was conducted using an online questionnaire distributed to dentists in Romania. The data collection period was from November 2025 to February 2026. A convenience sample was recruited through professional dental associations, social media groups, and direct email invitations. Inclusion criteria for participants were a valid license to practice dentistry in Romania and active performance of direct adhesive restorations. Out of the initial responses, 372 valid questionnaires were included in the final analysis. Although the questionnaires met the inclusion criteria, some item-level missing responses were present for specific variables. Given the recruitment strategy, the sample should be considered a convenience sample rather than representative of the entire population of dentists practising in Romania. To ensure data integrity, the online survey platform was configured to limit each participant to one submission, preventing duplicate entries.

The use of a convenience sampling strategy may have introduced selection bias, particularly toward younger and more digitally active practitioners, given the recruitment channels (social media and online distribution). This is reflected in the relatively high proportion of early-career respondents. Consequently, the findings should be interpreted with caution, as they may not fully represent the broader population of Romanian dentists, particularly those with extensive clinical experience or limited engagement in online professional networks.

### 2.2. Questionnaire

A comprehensive 115-item questionnaire was developed and pilot-tested for clarity and relevance. The questionnaire was specifically developed for this study, drawing on a comprehensive review of the existing literature on adhesive dentistry, previously published survey-based studies, and current clinical guidelines. Particular attention was given to ensuring that the items reflected contemporary concepts in adhesive systems and clinical decision-making. The initial version of the questionnaire was pilot-tested on a small group of practising dentists (*n* = 25) to assess clarity, relevance, and comprehensibility. Minor modifications were made in response to their feedback to improve wording and reduce ambiguity. However, the questionnaire was not formally validated as a standardised instrument prior to this study. The final instrument consisted of 115 items organised into five thematic sections, designed to capture demographic characteristics, clinical practices, knowledge of modern adhesive concepts, and attitudes toward continuing education and decision-making. The instrument was structured into five main sections:

I. Demographics and Professional Practice: Gender, year of graduation, academic degree, type of practice, years of clinical experience, and patient volume.
II. Clinical Adhesive Protocols: Preferred adhesive approach (ER, SE, selective etch), application times for etchants and adhesives, and moisture control techniques.
III. Knowledge of Modern Adhesive Concepts: A series of questions assessed the familiarity with and clinical application of eight key technologies, including bioactive fillers, MMP inhibitors, hydrolytic stability testing, and the role of 10-MDP.
IV. Continuing Education and Decision-Making: Primary sources for obtaining new information, frequency of attending continuing education courses, and the perceived importance of various factors (e.g., scientific evidence, cost, ease of use) when selecting an adhesive system.
V. Attitudes and Perceptions: Opinions on the adequacy of university education and the influence of manufacturers on clinical decisions.

### 2.3. Variables

For the purposes of this study, a knowledge score was computed for each participant based on their responses to the eight questions on modern adhesive technologies (items 95–102). The 8-item knowledge scale was derived from a predefined subset of questionnaire items (items 95–102) specifically designed to assess key contemporary concepts in adhesive dentistry, including bioactive materials, MMP inhibitors, hydrolytic stability, and the role of 10-MDP. These items were selected a priori based on their clinical relevance and representation in the current scientific literature. A value of 2 was assigned for ‘Know and apply’, 1 for ‘Know, but do not apply’, and 0 for ‘Do not know’, resulting in a total possible score from 0 to 16. The internal consistency of the 8-item knowledge scale was assessed using Cronbach’s alpha coefficient (α); a threshold of α ≥ 0.70 was considered acceptable [11]. The primary outcome variables were this knowledge score and the self-reported preferred adhesive approach. Predictor variables included the respondent’s graduation cohort (grouped as Group A: 1990–1997; Group B: 1998–2005; Group C: 2006–2013; Group D: 2014–2024), years of clinical experience, primary sources of information, gender, type of practice, and frequency of continuing education attendance. For participants who did not report their year of graduation (*n* = 76, 20.4%), the graduation year was estimated based on reported age, using a standardised assumption of 24 years as the typical age at graduation from dental school in Romania. This approach was adopted to retain these respondents in cohort-based analyses and to avoid unnecessary data loss. Although this method introduces a degree of approximation, the assumption is consistent with the structure of dental education in Romania and is unlikely to substantially affect the overall distribution of graduation cohorts. Nevertheless, this imputation may introduce minor misclassification and is acknowledged as a limitation of the study.

### 2.4. Statistical Analysis

The statistical analyses were performed using RStudio (version 4.2.2, USA) with the Tidyverse and Stats packages. Descriptive statistics (frequencies, percentages, means, standard deviations [SD], medians, and interquartile ranges [IQR]) were calculated to summarise the data. The internal consistency of the 8-item knowledge scale was evaluated using Cronbach’s alpha (α), with item-level analysis including corrected item–total correlations and α-if-item-deleted values. The Chi-square (χ^2^) test was used to investigate associations between categorical variables, such as graduation cohort and preferred adhesive approach. The Kruskal–Wallis H test was used to compare knowledge scores across graduation cohorts as the distributions were not normal. To identify independent predictors of the outcomes of interest, three multivariate models were constructed. First, a multiple linear regression analysis was performed with the knowledge score as the dependent variable and the following independent variables: gender, clinical experience (ordinal), frequency of reading indexed scientific articles, frequency of attending continuing education, perceived adequacy of university education, and university-based practice. Second, a binary logistic regression was conducted to identify predictors of high rubber dam use (≥50% of restorations), with gender, clinical experience, knowledge score, continuing education frequency, and university-based practice as independent variables; results were expressed as odds ratios (ORs) with 95% confidence intervals (CIs). Third, a multinomial logistic regression was performed with the preferred adhesive approach (ER, SE, selective etch, or variable) as the dependent variable and gender, clinical experience, knowledge score, and continuing education frequency as predictors. Model fit was assessed using the likelihood ratio test, Nagelkerke/McFadden pseudo-R^2^, and the Hosmer–Lemeshow test where appropriate. Multicollinearity was assessed using variance inflation factors (VIFs) with values < 5. A *p*-value of less than 0.05 was considered statistically significant for all tests.

Missing data were handled using a complete-case approach for each analysis. Item-level missing responses were not imputed and were treated as missing values; analyses were performed using the available data for each variable. The level of missingness was relatively low for most variables and is unlikely to have substantially affected the overall sample size in multivariate analyses.

### 2.5. Ethical Considerations

The study was conducted in accordance with the Declaration of Helsinki. The research protocol was approved by the Institutional Ethics Committee (No. 97/28.11.2025). All participants were informed of the study’s objectives, assured of their anonymity and the confidentiality of their data, and provided digital informed consent before completing the questionnaire.

## 3. Results

### 3.1. Demographic and Professional Profile of Respondents

A total of 372 dentists completed the survey. The demographic and professional characteristics of the sample are summarised in Table 1. The majority of respondents were female (73.7%). Of the 372 participants, 76 (20.4%) did not initially report their graduation year; for these respondents, the graduation year was imputed based on reported age (see Section 2.3), and they were subsequently reassigned to the corresponding graduation cohorts for all analyses. Following imputation, the largest cohort was Group D (2014–2024), representing 52.1% of the total sample, followed by Group B (1998–2005) at 26.1%. Most participants were general dental practitioners (72.8%), with a significant proportion working in private individual practice (37.6%). In terms of clinical experience, 39.2% had been in practice for 1–5 years.

### 3.2. Self-Reported Clinical Practices in Adhesive Dentistry

**Figure 1:**
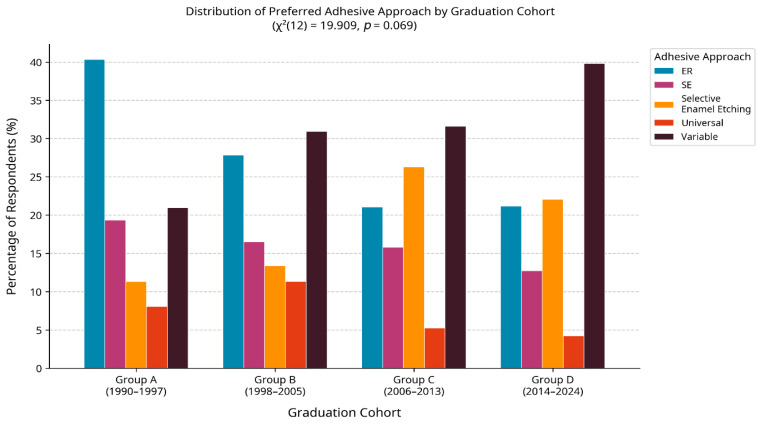
Distribution of preferred adhesive approaches among Romanian dentists (*n* = 372). The category “Variable” reflects respondents who reported adapting their adhesive strategy depending on the clinical situation.

When asked about their most frequently used adhesive approach, 34.4% of dentists reported that their choice varies depending on the clinical situation (Figure 1). The classic ER approach remains a popular choice, being the primary method for 28.5% of respondents. SE and selective etch were each preferred by 15.1% of participants. Universal adhesives, used in either ER or SE mode, were explicitly mentioned as the primary choice by a smaller fraction but were identified as the most commonly used system type overall (Table 2).

Regarding specific techniques, selective etch was reportedly practised in the majority of cases (>75%) by 40.9% of the dentists surveyed, indicating a widespread adoption of this evidence-based protocol to enhance enamel bonding for SE and universal systems (Figure 1).

### 3.3. Knowledge of Modern Adhesive Concepts

The 8-item knowledge scale demonstrated good internal consistency, with a Cronbach’s alpha of 0.873 (95% CI: 0.815–0.917), well above the a priori threshold of 0.70. Corrected item–total correlations ranged from 0.433 (MMP inhibitors) to 0.775 (“smart” adhesives), and the α-if-item-deleted analysis indicated that removal of any single item would not meaningfully improve the scale’s reliability (range: 0.840–0.878). The overall knowledge score, calculated on a scale of 0 to 16, had a mean of 7.51 (SD = 2.99) and a median of 8.0 (IQR = 6.0–9.0), suggesting a moderate level of familiarity with the assessed concepts. A significant finding was the proportion of uncertainty regarding the composition of the preferred adhesive system, with 14.8% of respondents explicitly reporting that they did not know whether their system contained the 10-MDP monomer.

**Figure 2:**
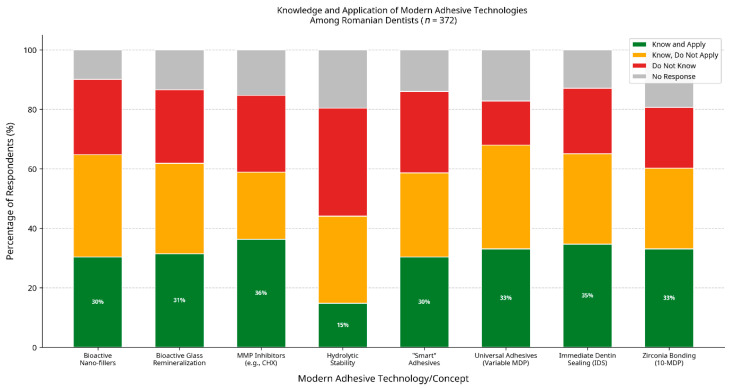
Knowledge and application of modern adhesive technologies among Romanian dentists (*n* = 372). The “knowledge-practice gap” is represented by the orange segment (Know, Do Not Apply).

Table 2 presents the distribution of knowledge and application for eight modern adhesive technologies (Figure 2). A consistent pattern of a “knowledge-practice gap” emerged. For instance, while a combined 58.9% of dentists were aware of MMP inhibitors, only 36.3% reported applying this knowledge clinically. The largest knowledge deficit was observed for the concept of hydrolytic stability testing (thermocycling), with 36.3% of participants unaware of it.

### 3.4. Sources of Information and Attitudes

**Figure 3:**
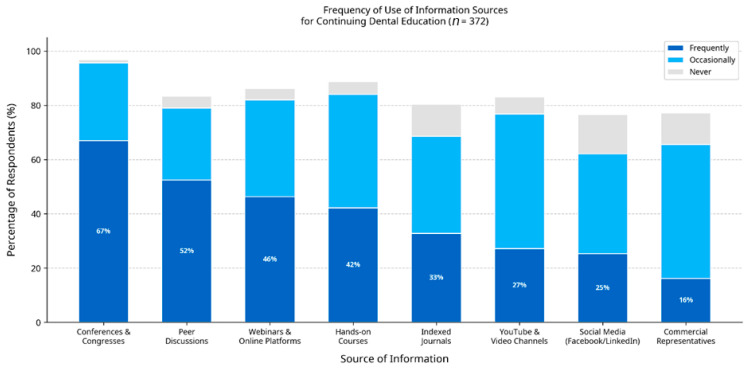
Frequency of use of information sources for continuing dental education among respondents (*n* = 372). Sources are ordered by frequency of use (descending).

The most frequently utilised sources for continuing education were professional conferences and congresses (66.9%), discussions with colleagues (52.4%), and online webinars (46.2%) (Figure 3). Notably, indexed scientific articles were a frequent source of information for only 32.8% of dentists. A majority of respondents (51.6%) felt that their undergraduate education had only partially prepared them for using modern adhesive systems, indicating a perceived need for supplementary training. Furthermore, 50.5% acknowledged a moderate influence of dental manufacturers on their clinical decisions.

### 3.5. Association Analyses

No statistically significant association was found between the dentists’ graduation cohort and their preferred adhesive approach (χ^2^(12) = 19.909, *p* = 0.069). Similarly, the Kruskal–Wallis test revealed no significant differences in median knowledge scores across the four graduation groups (H = 5.918, *p* = 0.116), suggesting that clinical experience alone may not be a primary determinant of knowledge regarding these specific modern concepts. No significant association was found between the frequency of reading scientific articles and higher knowledge scores (χ^2^(1) = 0.116, *p* = 0.733).

### 3.6. Multivariate Analyses

To move beyond bivariate associations and identify independent predictors, three multivariate models were constructed. Variance inflation factors for all models were below 2.0, indicating no problematic multicollinearity among predictors.

#### 3.6.1. Predictors of Knowledge Score (Multiple Linear Regression)

A multiple linear regression model was fitted with the knowledge score as the dependent variable and six predictors: gender, clinical experience, frequency of reading indexed scientific articles, frequency of continuing education (CE) attendance, perceived adequacy of university education, and university-based practice. The overall model was statistically significant (F(6, 365) = 3.663, *p* = 0.004) and explained approximately 5.7% of the variance in knowledge scores (adjusted *R*^2^ = 0.057). The only predictor that reached statistical significance was the frequency of continuing education attendance (β = 1.63, 95% CI: 0.22–3.04, *p* = 0.024), indicating that dentists who attended CE courses more frequently scored, on average, 1.63 points higher for each ordinal increase in CE frequency, after controlling for all other variables. Neither gender, clinical experience, frequency of reading scientific articles, perceived adequacy of university education, nor university-based practice were independently associated with the knowledge score (all *p* > 0.05).

#### 3.6.2. Predictors of Rubber Dam Use (Binary Logistic Regression)

A binary logistic regression was conducted to identify predictors of high rubber dam use (defined as ≥50% of direct restorations). The model included gender, clinical experience, knowledge score, CE frequency, and university-based practice as covariates. The overall model did not reach statistical significance (LR χ^2^ = 8.76, *p* = 0.119; pseudo-*R*^2^ = 0.120). Clinical experience was the only statistically significant predictor (OR = 0.550, 95% CI: 0.327–0.924, *p* = 0.024): for each higher clinical experience category, the odds of reporting high rubber dam use decreased by 45%. Gender showed a non-significant trend (OR = 0.380, 95% CI: 0.102–1.412, *p* = 0.148), with male dentists being less likely to report high usage. The knowledge score was not a significant predictor of rubber dam use (OR = 1.074, 95% CI: 0.919–1.255, *p* = 0.371).

#### 3.6.3. Predictors of Adhesive Approach (Multinomial Logistic Regression)

A multinomial logistic regression was performed with the preferred adhesive approach as the dependent variable (categories: ER, SE, selective etch, and variable, with variable as the reference category) and gender, clinical experience, knowledge score, and CE frequency as predictors. The overall model did not reach statistical significance (LR χ^2^ = 13.43, *p* = 0.339; pseudo-*R*^2^ = 0.082), indicating that the measured demographic and professional variables do not adequately explain the choice of adhesive approach. Clinical experience showed a marginal, non-significant trend toward predicting ER preference over a variable approach (β = –0.737, *p* = 0.097). No other predictor was statistically significant in any of the comparisons (all *p* > 0.05), confirming and extending the earlier bivariate findings that the choice of adhesive strategy is largely independent of the demographic and educational variables assessed in this study.

## 4. Discussion

This study provides a detailed snapshot of the current landscape of adhesive dentistry in Romania, moving beyond mere preferences to explore practitioners’ underlying knowledge and attitudes. Our findings indicate a high overall level of awareness and familiarity with universal adhesives in current clinical practice, with 68.0% of respondents reporting familiarity with and/or clinical use of these systems. This finding is consistent with both national and international trends and corroborates the observations reported by Olariu et al. [20], who similarly documented the popularity of universal adhesive systems among Romanian dentists. Internationally, the widespread adoption of universal adhesives has also been extensively reported, largely due to their clinical versatility, simplified application protocols, and reliable bonding performance across multiple substrates [21,22].

The central finding of our study is the presence of a measurable “knowledge–practice gap,” defined here as the discrepancy between dentists’ self-reported awareness of contemporary adhesive concepts and their reported clinical application of these concepts. Importantly, this gap should not be interpreted as a direct indicator of inappropriate or suboptimal clinical practice. In some cases, clinicians may follow evidence-based protocols without explicitly identifying or conceptualising the underlying scientific principles. Although many dentists reported awareness of advanced adhesive concepts, a substantial proportion did not translate this knowledge into clinical application [23,24]. For example, 29.3% of respondents reported being familiar with the concept of hydrolytic stability but not applying it in practice, while 34.9% were aware of the variable 10-MDP content in universal adhesives but did not incorporate this knowledge into their clinical decision-making. Rather than reflecting a lack of clinical competence, this discrepancy may reflect differences in how theoretical knowledge is internalised and translated into clinical decision-making. It may also be influenced by variations in educational exposure, confidence in emerging evidence, and perceived clinical relevance [25].

Particularly concerning is the finding that 14.8% of dentists reported being unaware of whether their preferred adhesive system contained 10-MDP. Given that the chemical bonding capacity provided by 10-MDP represents a cornerstone of the performance of most modern universal adhesives, this knowledge deficit is clinically relevant [26,27]. This suggests that some clinicians may use these materials without fully understanding their mechanism of action, potentially compromising outcomes in clinically demanding situations such as zirconia bonding or self-etch application on uncut enamel.

The study also provides valuable insight into the current ecosystem of continuing dental education in Romania. The greater reliance on conferences, webinars, and peer discussions, compared with indexed scientific literature (which is frequently used by only 32.8% of respondents), may partially contribute to the observed discrepancy between knowledge and implementation. Although these educational formats are valuable for sharing practical clinical guidance, they may not always provide the evidence-based depth needed to critically evaluate and implement emerging technologies. Moreover, the perception that undergraduate education was only partially adequate further supports the need for structured postgraduate training.

Contrary to our initial hypothesis, the bivariate analyses found no significant association between years of experience (as proxied by graduation cohort) and either the choice of adhesive system or the level of knowledge. The multivariate analyses added further nuance to this finding. In the multiple linear regression model, the frequency of continuing education attendance emerged as the only independent predictor of higher knowledge scores (β = 1.63, *p* = 0.024), whereas clinical experience, gender, reading indexed articles, perceived quality of university education, and university-based practice did not reach statistical significance. This suggests that the active, ongoing pursuit of professional development, rather than years of clinical exposure, academic training, or self-directed reading, is associated with higher levels of knowledge regarding modern adhesive concepts. Interestingly, the binary logistic regression for rubber dam use revealed an inverse association with clinical experience (OR = 0.550, *p* = 0.024), indicating that more experienced dentists are less likely to report high rubber dam utilisation. This finding is particularly noteworthy when compared with the existing literature. In the study by Mashyakhy et al., a high rubber dam usage rate of 71% was reported among participants, suggesting relatively high adherence to recommended isolation protocols [28]. In contrast, studies by Sanghvi et al. and Ibhawoh et al. reported substantially lower rates of rubber dam utilisation, highlighting significant variability in clinical practice across different professional and geographical settings [29,30]. These discrepancies may reflect differences in undergraduate training, access to continuing professional education, institutional culture, and clinicians’ perceptions regarding the necessity and practicality of rubber dam isolation in routine restorative procedures.

These findings align with global research indicating significant variation in the implementation of evidence-based adhesive and isolation measures across diverse geographical contexts. European studies have shown that rubber dams are used at moderate levels: about 35.6% of practitioners use them during restorative procedures and 71.1% during endodontic treatment [31]. This shows that there is still a gap between recommended protocols and what is done in everyday clinical practice. Conversely, research from the Middle East indicates even lower adherence rates among general practitioners, often attributed to time constraints, perceived procedural complexity, and inadequate instruction during undergraduate studies [32]. These international comparisons suggest that the divergence between evidence and practice observed in our cohort is not an isolated national issue but rather reflects a broader global challenge in integrating evidence-based restorative dentistry into everyday clinical practice [33].

This seemingly counterintuitive finding may reflect the persistence of established clinical routines that are resistant to change or, alternatively, evolving perceptions regarding the necessity of isolation over the course of longer professional careers. Furthermore, the multinomial logistic regression confirmed that the choice of adhesive approach was not significantly predicted by any of the demographic or educational variables examined (LR *p* = 0.339), suggesting that technique selection may be influenced by factors beyond those captured in the present study, such as hands-on training, personal clinical experience and outcomes, or the influence of peers and mentors. Taken together, these findings highlight structured continuing education as the main modifiable factor associated with improved knowledge in adhesive dentistry and suggest potential gaps in both undergraduate and postgraduate training.

Similar challenges related to professional training structures and workforce characteristics have also been reported in emerging economies, where variability in educational exposure and access to structured continuing education may influence clinical decision-making and adoption of modern technologies [34].

This study is not without limitations. First, the use of a convenience sample limits the representativeness of the findings. Although the sample size was relatively large (*n* = 372), the recruitment strategy does not allow us to conclude that the respondents are fully representative of the broader population of dentists practising in Romania. Recruitment through professional associations, social media groups, and direct email invitations may have preferentially attracted younger, more digitally active, and academically engaged practitioners. This is reflected in the predominance of early-career respondents in the sample, with 33.1% reporting less than one year of clinical experience and 39.2% reporting 1–5 years of practice. Consequently, the findings may overrepresent the perspectives, educational exposure, and clinical decision-making patterns of younger clinicians, while potentially underrepresenting senior practitioners and their established clinical routines. In addition, because no formally stratified sampling strategy was employed, potential regional, institutional, or speciality-related imbalances cannot be excluded. Therefore, caution is warranted when extrapolating these findings to the entire population of Romanian dentists. Furthermore, the self-reported nature of the data introduces the possibility of recall and social desirability bias, as participants may have overestimated their knowledge or adherence to recommended clinical practices.

Despite these limitations, the large sample size, the good internal consistency of the knowledge scale (α = 0.873), and the questionnaire’s detailed, multifaceted nature provide a valuable and original contribution to the literature on this topic. In addition, item-level missing responses were observed for certain questionnaire items, with a non-response rate of approximately 15% for some knowledge-related variables. Although a complete-case analysis was applied, this may have introduced bias if the missingness was not completely random.

## 5. Conclusions

This study highlights a clear discrepancy between dentists’ awareness of modern adhesive concepts and their consistent clinical application. While familiarity with universal adhesives is high, clinically relevant knowledge gaps persist, particularly regarding key components such as 10-MDP. Continuing education frequency emerged as the only independent predictor of higher knowledge levels, whereas clinical experience was inversely associated with rubber dam use. These findings emphasise the need for targeted, evidence-based continuing professional education to support the effective translation of scientific knowledge into daily clinical practice.

Given the exploratory nature of the study, the convenience sampling strategy, the self-reported nature of the data, and the presence of item-level missing responses and age-based imputation, these findings should be interpreted with caution and should not be directly generalised to the entire Romanian dental workforce without further confirmatory studies using representative sampling methods.

## Figures and Tables

**Table 1:** Demographic and professional characteristics of the study participants (*n* = 372).

Characteristic	Category	*n*	%
**Gender**	Female	274	73.7%
	Male	98	26.3%
**Graduation Cohort**	Group A (1990–1997)	62	16.7%
	Group B (1998–2005)	97	26.1%
	Group C (2006–2013)	19	5.1%
	Group D (2014–2024)	194	52.1%
**Primary Role**	General Dentist	271	72.8%
	Specialist	59	15.9%
	Resident/Primary	25	6.7%
	PhD	17	4.6%
**Type of Practice**	Private Individual Practice	140	37.6%
	Private Clinic (multi-practitioner)	99	26.6%
	Mixed (Public + Private)	91	24.5%
	University Clinic	42	11.3%
**Clinical Experience**	1–5 years	146	39.2%
	<1 year	123	33.1%
	11–20 years	47	12.6%
	6–10 years	31	8.3%
	>20 years	25	6.8%

**Table 2:** Knowledge and application of modern adhesive technologies among respondents (*n* = 372).

Technology/Concept	Know and Apply (*n*, %)	Know, Do Not Apply (*n*, %)	Do Not Know (*n*, %)	No Response (*n*, %)
Bioactive Nano-fillers	113 (30.4%)	128 (34.4%)	94 (25.3%)	37 (9.9%)
Bioactive Glass Remineralization	117 (31.5%)	113 (30.4%)	92 (24.7%)	50 (13.4%)
MMP Inhibitors (e.g., CHX)	135 (36.3%)	84 (22.6%)	96 (25.8%)	57 (15.3%)
Hydrolytic Stability (Thermocycling)	55 (14.8%)	109 (29.3%)	135 (36.3%)	73 (19.6%)
“Smart” Adhesives	113 (30.4%)	105 (28.2%)	102 (27.4%)	52 (14.0%)
Universal Adhesives (variable MDP)	123 (33.1%)	130 (34.9%)	55 (14.8%)	64 (17.2%)
Immediate Dentin Sealing (IDS)	129 (34.7%)	113 (30.4%)	82 (22.0%)	48 (12.9%)
Zirconia Bonding (10-MDP)	123 (33.1%)	101 (27.2%)	76 (20.4%)	72 (19.4%)

## Data Availability

The original contributions presented in this study are included in the article. Further inquiries can be directed to the corresponding authors.
